# Current status of hand-foot-and-mouth disease

**DOI:** 10.1186/s12929-023-00908-4

**Published:** 2023-02-24

**Authors:** Peiyu Zhu, Wangquan Ji, Dong Li, Zijie Li, Yu Chen, Bowen Dai, Shujie Han, Shuaiyin Chen, Yuefei Jin, Guangcai Duan

**Affiliations:** 1 Department of Epidemiology, College of Public Health, Zhengzhou University, Zhengzhou, 450001 China; 2 Academy of Medical Science, Zhengzhou University, Zhengzhou, 450001 Henan China

**Keywords:** Hand-foot-and-mouth disease, Epidemiological characteristic, Innate immune response, Sequelae, Treatment

## Abstract

Hand-foot-and-mouth disease (HFMD) is a viral illness commonly seen in young children under 5 years of age, characterized by typical manifestations such as oral herpes and rashes on the hands and feet. These symptoms typically resolve spontaneously within a few days without complications. Over the past two decades, our understanding of HFMD has greatly improved and it has received significant attention. A variety of research studies, including epidemiological, animal, and in *vitro* studies, suggest that the disease may be associated with potentially fatal neurological complications. These findings reveal clinical, epidemiological, pathological, and etiological characteristics that are quite different from initial understandings of the illness. It is important to note that HFMD has been linked to severe cardiopulmonary complications, as well as severe neurological sequelae that can be observed during follow-up. At present, there is no specific pharmaceutical intervention for HFMD. An inactivated Enterovirus A71 (EV-A71) vaccine that has been approved by the China Food and Drug Administration (CFDA) has been shown to provide a high level of protection against EV-A71-related HFMD. However, the simultaneous circulation of multiple pathogens and the evolution of the molecular epidemiology of infectious agents make interventions based solely on a single agent comparatively inadequate. Enteroviruses are highly contagious and have a predilection for the nervous system, particularly in child populations, which contributes to the ongoing outbreak. Given the substantial impact of HFMD around the world, this Review synthesizes the current knowledge of the virology, epidemiology, pathogenesis, therapy, sequelae, and vaccine development of HFMD to improve clinical practices and public health efforts.

## Introduction

**Fig. 1:**
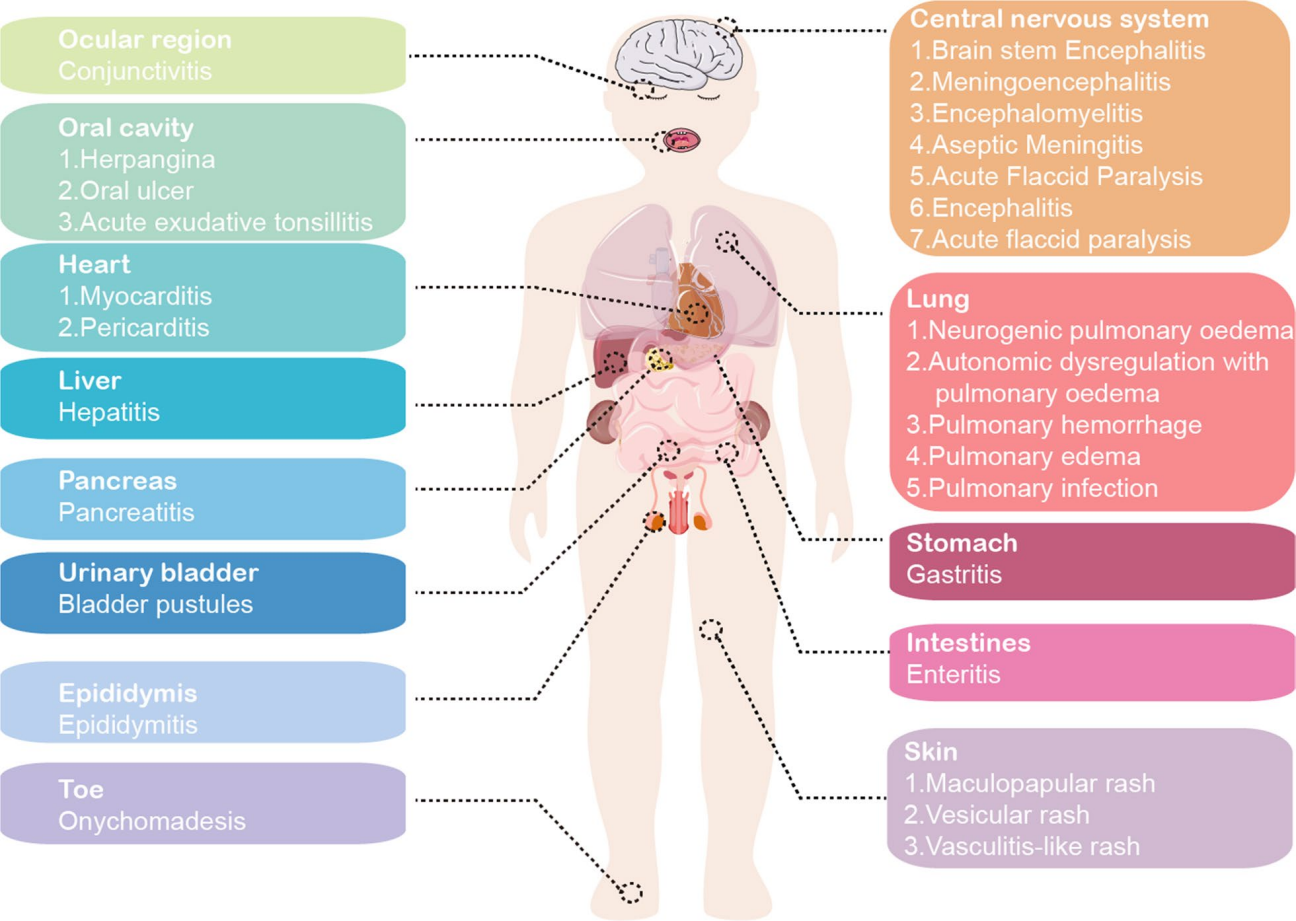
**Complications and sequelae of HFMD**

As early as 1957, the characteristic symptoms of fever, vesicular rash on hands and feet caused by Coxsackievirus (CV), primarily CVA16, was first reported in Toronto [1, 2]. In 1959, “hand-foot-and-mouth disease (HFMD)” was initially used to name a disease with essentially the same symptoms as described by Robinson et al. [3]. Over the past few decades, HFMD outbreaks caused by Enterovirus A71 (EV-A71), CVA16, CVA6 and Echoviruses (Echo) were reported frequently around the world [4]. EV-A71, which was first isolated from a child with meningitis in 1969, has also caused widespread outbreaks of HFMD throughout much of the Asia–Pacific region [5]. The disease was generally mild and lasted less than a week in most cases, characterized by fever, a blister-like rash on the hands and feet, and oral ulcers caused by ruptured blisters in the mouth [3]. However, quite a few patients experience fatal neurological or cardiopulmonary complications. Furthermore, recent follow-up studies have shown that severe neurological sequelae may occur in severely recovered patients (Fig. 1) [6–8]. Therefore, HFMD has become a significant concern for public health throughout the Asia–Pacific region and beyond. The discovery of tomato flu, a HFMD-like illness caused by enterovirus, in India has brought renewed attention to HFMD outbreaks [9]. This Review focuses on summarizing the current findings regarding HFMD in regards to virology, epidemiology, pathogenesis, and vaccine development in order to better inform clinical practice and public health initiatives.

### Etiological characteristics of HFMD

HFMD is caused by Human enteroviruses (EVs) that are members of the *Enterovirus* genus of the *Picornaviridae* family [10]. EVs were initially classified into Poliovirus (PV), Echo, CV-A and B, and emerging EVs. Since 1999, EVs have been divided into four categories of Enterovirus A, B, C, and D, in the light of their molecular, biological, and genetic characteristics. Nowadays, over 100 EVs have been reported worldwide [10]. Table 1 lists various pathogens associated with HFMD outbreaks [11]. In the past, EV-A71 and CVA16 were the most frequently reported causes of HFMD prior to 2005. Currently, other EVs such as CVA6 and CVA10 are responsible for a significant proportion of HFMD cases and outbreaks [12, 13]. Although CVB1-5 associated with HFMD had also been mentioned in several reports, the impact of these pathogens on HFMD is still expanding [14, 15].

**Table 1:** EVs associated with HFMD

Species	Associated Enterovirus serotypes
EV-A	CVA2, CVA4, CVA5, CVA6, CVA7, CVA8, CVA10, CVA12,CVA13, CVA16
	EV-A69, EV-A71
EV-B	CVA9, CVB1, CVB2, CVB3, CVB4, CV-B5
	E-3, E-4, E-5, E-6, E-7, E-9, E-11, E-14, E15, E16, E-18, E-19, E-21, E-30, EV-B84
EV-C	CVA1, CVA19, CVA21, CVA22, CVA24, EV-C99

**Fig. 2:**
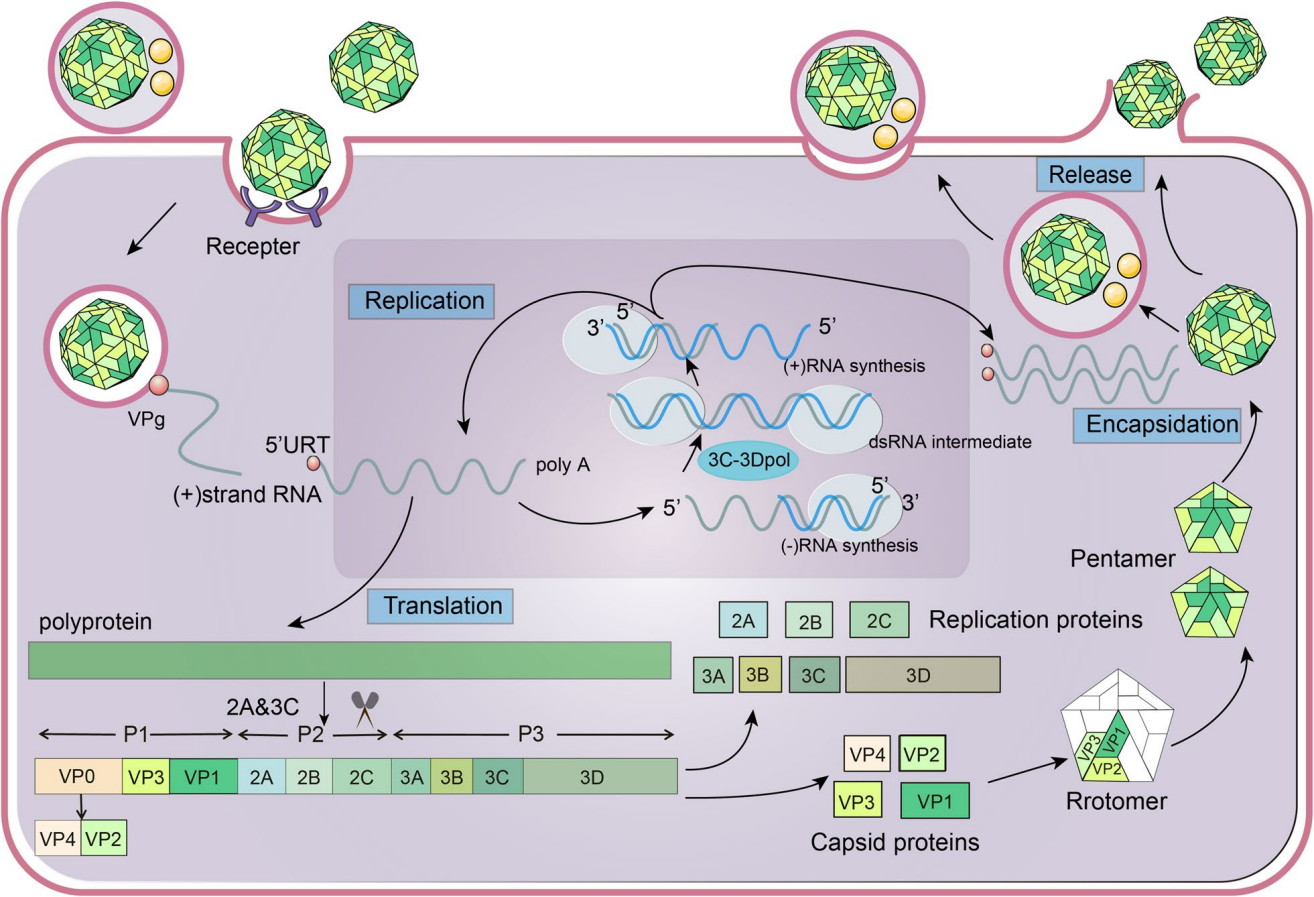
**The life cycle of Enterovirus.** Enterovirus (EVs) enter the host cells by binding to receptors or by exosome-mediated endocytosis and release positive-strand RNA. The RNA undergoes transcription and translation after being covalently linked to the viral protein VPg (3B). The translated polyprotein is hydrolyzed by various proteases into 10 separate major proteins, including VP0, VP1, VP3, 2A-C, 3A-D, where VP0 is subsequently hydrolyzed to VP2 and VP4. VP1-4 are assigned to participate in the assembly of viral protein coats, while 2A-C, 3A-D are directed to participate in the replication of viral genetic material. Finally, the viral RNA and coat are assembled and processed into mature viruses, which are then co-packaged with host organelle decomposers in vesicles and secreted out of the cell, or directly released by exocytosis

The viral particle of EVs is symmetrical icosahedron composed of 60 subunits of coat protein and a single-stranded RNA genome (7.5 kb) of positive polarity (Fig. 2) [16]. The open reading frame (ORF) of the viral genome encodes 2194 amino acids (Fig. 2), and the 3′ untranslated region UTR (3'UTR) is followed by a poly-A tail of variable length. The protein encoded by the viral genome mainly include three regions: P1, P2, and P3, of which P1 encodes four structural proteins, VP1-VP4, and the P2 and P3 encode seven non-structural proteins, 2A-2C and 3A-3D, respectively [17, 18]. VP1-VP4 are further involved in virion capsid assembly [17]. Although VP1, VP2 and VP3 are arranged on the outer side of the capsid, VP1 is the main antigen-binding site [19]. Thus, VP1 is a suitable candidate for major serotyping and vaccine development and has been widely used as a target gene for EVs molecular research [20]. Moreover, Physico-chemical characteristics of EVs include resistance to organic solvents such as ether and chloroform and low temperature conditions, and sensitivity to high temperature, chlorinated disinfectants, formaldehyde and ultraviolet etc. [10].

## Epidemiological characteristics

### Epidemic process and influencing factors

#### Clinical features

The criteria for diagnosing of HFMD, which are widely accepted at present, primarily rely on the patient’s epidemiological history, symptoms additional tests to determine the cause or presence of the disease [21]. This includes examination of the patient's age, the timing of onset, gathering place, and if they had direct or indirect contact with HFMD infections before the onset of the disease [21]. The incubation period of HFMD is mostly 2–10 days, with an average of 3–5 days. The progression of HFMD be divided into 5 stages (rash, neurological dysfunction, early stage of cardiopulmonary failure, cardiopulmonary failure, recovery), and most cases generally only experience the first stage and recover within a week [22]. Clinically, most cases have fever accompanied by rash on hands, feet, mouth, and buttocks [21]. The prevention in patients with severe HFMD depends on the timely and accurate identification of danger signs in the disease progression [21]. The following 7 indicators are considered as risk factors of HFMD severity: (1) high fever; (2) nervous system involvement; (3) abnormal respiratory rate and rhythm; (4) circulatory dysfunction; (5) increased white blood cell count; (6) increased blood glucose; (7) increased blood lactate [21, 23]. In some cases of HFMD, the rash is atypical such as a single site or a maculopapular rash only. Most cases usually need to be differentiated from papular urticaria, chickenpox, herpes zoster, rubella, and herpes simplex caused by other diseases [21]. In addition, neurogenic pulmonary edema (PE) should be distinguished from pneumonia. Clinical samples (pharyngeal swabs, stool or anal swabs, blood, blister fluid, cerebrospinal fluid, etc.) are tested through RT-PCR, virus isolation, neutralizing antibody testing [21]. Subsequently, clinicians diagnosed the suspected patient as a confirmed case of HFMD based on epidemiological history, clinical manifestations, and laboratory nucleic test [24].

#### Source of infection

Human is usually considered to be the only reservoir of human EVs, and both cases and asymptomatic infections are the sources of HFMD infection. The virus can be detected in the pharynx and feces of infected individuals in the days before the onset of illness, and is usually most contagious within a week after the onset of symptoms. Therefore, the presence of asymptomatic infections and those in the incubation period may complicate efforts to prevent and control HFMD.

#### Routes of transmission

Currently, the fecal–oral transmission and contact are considered as the primary transmission routes of HFMD. The potential transmission routes of aerosols and respiratory tract have been proposed based on some animal studies [25]. Further research is required to fully understand and confirm the transmission routes of both aerosol and droplet in human population.

#### Herd susceptibility

As a common childhood infectious disease, HFMD primarily occurs in children under 5 years old [26], although HFMD has also been reported in adults [27]. Children are highly susceptible to the EVs due to immature immune system and clustering at the pre-kindergarten stage [28]. China has implemented kindergarten closures to block the transmission of coronavirus disease (COVID-19), indirectly reducing the incidence of HFMD and preventing HFMD outbreaks [29, 30]. In addition to reducing the clustering of susceptible populations and enhancing individual protection, “herd” immunity through vaccination is more effective in reducing population susceptibility. The urban area with high EV-A71 transmission in China initiated vaccination with inactivated EV-A71 vaccine, a dramatic decline in EV-A71-associated HFMD incidence was observed [31]. Patients, both dominant and recessive infections caused by EVs, can acquire specific immunity, and the neutralizing antibodies can be retained in the body for a long time. EVs can stimulate stronger immune response, but there is almost no cross-immunity between different serotypes. Consequently, multivalent vaccines are urgently needed to further improve herd immunity.

#### Spectrum of infection

HFMD is always considered as a type of self-limiting infectious disease, and most patients with mild symptoms recover within 1 ~ 2 weeks. Large-scale observational studies showed that There are 5 different outcomes of HFMD: asymptomatic (12.7%), mild (86.2%), severe and critical (1.1%), death (0.03%) [24], [32].

#### Natural factors

Both high and low temperatures were associated with the incidence of HFMD [33]. For example, CVA6 outbreaks usually occur in winter [34]. Precipitation and humidity could provide the necessary water environment and aerosols for virus survival, and protect the virus from harmful factors such as temperature, salinity, and pH [35]. The intensity of UV exposure time also affected the incidence of HFMD [36]. Also, the terrain dominated by mountains or hills with lower atmospheric pressure affects the incidence of HFMD [37]. Recently, a new attention has been attracted to the impact of air pollution on HFMD epidemic. Yu et al. found that exposure to environmental particulate matter increases the risk of children developing HFMD. They believed that these particulates may facilitate virus transmission through airborne infections and that high wind speeds further contribute to the spread of virus-carrying particles [28]. Besides, ozone might affect infectious diseases by inhibiting the ability of virus to exist in the external environment [28].

#### Socioeconomic factors

Socioeconomic factors are also closely related to the epidemic of HFMD. The incidence of HFMD in urban residents, transportation hub cities, and economically developed areas compared to rural area, this is due to the higher population density and mobility in these areas [26, 38]. Health regulations and large-scale vaccination in educational settings promulgated by the state or government at all levels significantly reduced the incidence of HFMD [28]. The lack of medical insurance coverage and ethnic minorities are all risk factors for HFMD [26]. Rural residents and poverty are both risk factors for HFMD severity, which may be caused by poor sanitation, lower educational attainment, and lower economic status [26]. Furthermore, factors such as being raised at home, having a larger family size, and poor hand hygiene are associated with a higher risk of HFMD transmission [39, 40]. Short interval from onset to hospitalization, hospitalization in a high-level hospital, and treatment by more experienced doctor are protective factors for HFMD severity [41]. Lack of breastfeeding in children with lower immune status may lead to HFMD severity [42, 43]. Extended gatherings of children in schools or daycare centers can facilitate the transmission of HFMD, while taking appropriate breaks during vacation time can serve as a protective measure against it [44].

### The four main EV serotypes causing HFMD outbreaks

#### HFMD of outbreaks caused by EV-A71

**Fig. 3:**
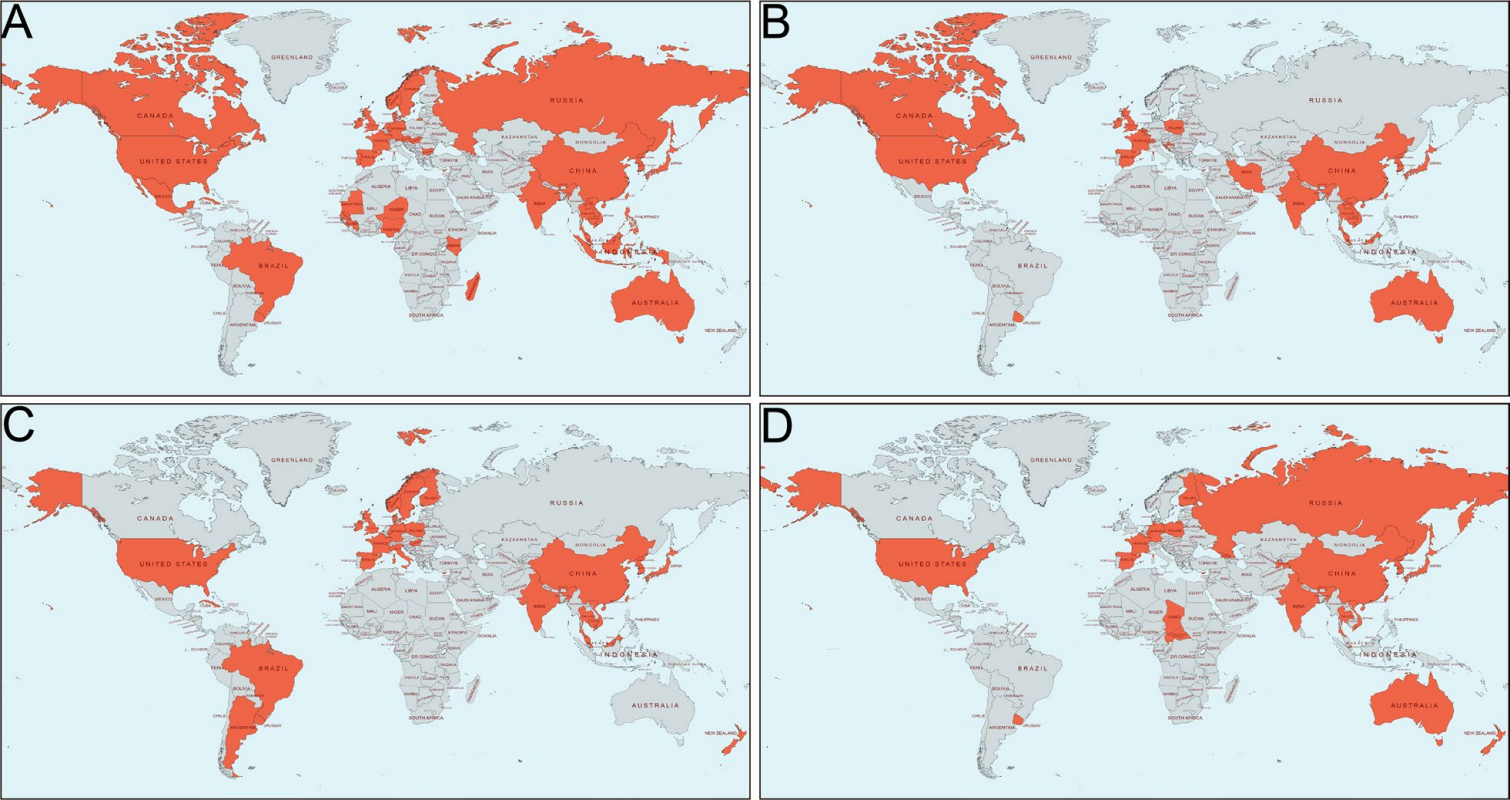
**Distribution of patients with HFMD in the world.** **A** EV-A71; **B** CVA16; **C** CVA6; **D** CVA10. Areas marked in orange indicate that EV-A71/CVA16/CVA6/CVA10 epidemic have been reported

The EV-A71 strain was first isolated in California in 1969 [5]. During 1970–1990, HFMD outbreaks caused by EV-A71 occurred frequently in the United States [45–47]. In the European, including Sweden [48], Bulgaria [49], Hungary [50], and the Netherlands [51], outbreaks of HFMD related to EV-A71 have been monitored. Japan [52], Brazil [53] and Australia have also reported a large number of cases of aseptic meningitis and brainstem encephalitis associated with EV-A71. At the end of the twentieth century, EV-A71 activity increased dramatically throughout the Western Pacific region. In 1997, a large outbreak of HFMD caused by EV-A71 strain in Malaysia resulted in 41 fatalities [54]. Next year, Taiwan (China) reported 100,000 cases of HFMD mainly caused by EV-A71, including 400 severe cases and 78 deaths [55]. During the period from 2008 to 2014, a total of 10,717,283 cases (3046 deaths) were reported in China, and the fatality rate was 0.03%. Among survivors, the incidence increased from 37.6/100,000 (2008) to 139.6/100,000 (2013) and had a peak in 2012 at 166.8/100,000. In 2011–2012, a large-scale EV-A71 outbreak in Vietnam resulted in more than 200,000 hospitalizations and 207 deaths [56]. In 2012, EV-A71 infection killed at least 54 children with severe encephalitis in Cambodia (26,690,000). In addition, Russia [57], South Korea [58], Singapore [59], Thailand [60, 61] and Philippines [62] have also experienced large-scale EV-A71 outbreaks. Recently, European countries such as Denmark [63], France [64], Germany [65], Spain [66] and Poland [67] also reported sporadic cases (Fig. 3).

#### HFMD of outbreaks caused by CVA16

CVA16 was the main pathogen of HFMD outbreaks in England in 1959 and 1994 [3, 68]. There were also CVA16 outbreaks in the United States in 1964 and 1968 [69, 70]. CVA16 infection was also responsible for the 1991 outbreak of HFMD in Sydney, Australia [71]. Subsequently, the Asian-Pacific region includes China [72, 73], Japan [74], India [75, 76], Taiwan (China) [77], Vietnam [78], Singapore [79, 80], and Spain in Europe [81] reported CVA16 outbreaks. Currently, CVA16 pathogens are frequently detected together with CVA6 and CVA10 [43, 82–84].

#### HFMD outbreaks caused by CVA6

In recent years, the pathogenic spectrum of HFMD has changed with inoculation of EV-A71 vaccines, especially in China. From 2016 to 2018, the proportion of EV-A71 and CVA16 positive was 8.9%, 5.2%, respectively, while the proportion of other EVs was 60.6% among 3559 HFMD cases in Hangzhou, China [85]. Since an outbreak of HFMD caused by CVA6 in Finland in 2008, CVA6 is responsible for a series of HFMD outbreaks in Europe, Northern America, and Asia [86]. In recent years, HFMD outbreaks caused by CVA6 have occurred in the United States [87, 88], Spain [81], Hungary [89], France [84] and the United Kingdom (Fig. 3) [90]. EVs were detected in 2228 HFMD patients in Vietnam from 2008 to 2017, and CVA6 accounted for 28.4%, only second to EV-A71 (31.7%). However, the large-scale HFMD outbreak in Thailand in 2012 showed that in 672 HFMD cases, 221 (32.9%) were caused by CVA6 [91]. In 2011, the National Epidemiological Surveillance System of Infectious Diseases of Japan reported an increase rate of CVA6 detection in HFMD cases [92]. In the massive HFMD epidemic that occurred in Japan in 2017, CVA6 was the primary pathogen responsible for the illness of 6,173 patients [93]. In addition, Singapore [94], New Zealand [95] and Malaysia [96] have also reported HFMD CVA6 is the dominant strain of HFMD outbreaks. Since 2013, CVA6-associated HFMD has been on the rise in parts of China [97–100]. Unlike HFMD caused by other EVs serotypes, CVA6-associated HFMD presents a more severe and extensive rash, and is also characterized by a higher incidence in adults, winter onset, and a tendency to shed arm after recovery [34, 101, 102].

#### HFMD of outbreaks caused by CVA10

The prototype strain of CVA10, Kowalik (GenBank ID: AY421767), was isolated in the United States in 1950 [103]. In May 1961, the CVA10 strain was also isolated in 40 children with HFMD reported in New Zealand [104]. The first detailed outbreak of CVA10 occurred in Japan between July 1981 and January 1982. Thirty seven clinical HFMD cases were examined for virology and serology, and CVA10 was detected in 18 cases [105]. Subsequently, Asia, Europe, Africa, and Oceania successively reported HFMD associated with CVA10 co-transmitted with CVA6 (Fig. 3). In 2008, clinical specimens were obtained from 317 HFMD cases in Finland, including adults and children, and the proportion of CVA10 (28%) was only second to CVA6 (71%) [86]. The HFMD epidemic surveillance in Singapore in 2008 showed that the detection rate of CVA10 (11.8%) ranked third, followed by CVA6 (23.5%) and EV-A71 (21.6%) [94]. A French sentinel surveillance data study conducted in 2010 reported that CVA10 (39.9%) was the leading serotype responsible for HFMD [106]. In Asia, CVA10 was also the most common pathogen in HFMD cases monitored in Korea in 2008 [107]. In a prospective cohort study in 2016, a higher proportion of CVA10 was detected in HFMD cases [43]. There are different levels of CVA10 detection rates in HFMD patients across China. CVA10 (25%), CVA6 (29.8%), and CVA16 (32.5%) were the most common serotypes [108] in HFMD patients in Guangdong Province in 2018. In Xiamen, from 2009 to 2015, the proportion of CVA10 in cases of HFMD was not particularly high (1.08–7.09%). However, the detection rate of CVA10 in severe HFMD cases was significantly higher than in previous years [109]. From 2016 to 2020, a total of 9952 sporadic HFMD cases in Shanghai were collected and CVA10 was the fourth major epidemic pathogens, with a total positive rate of 2.78% [110].

### Genetic evolution of EVs

**Fig. 4:**
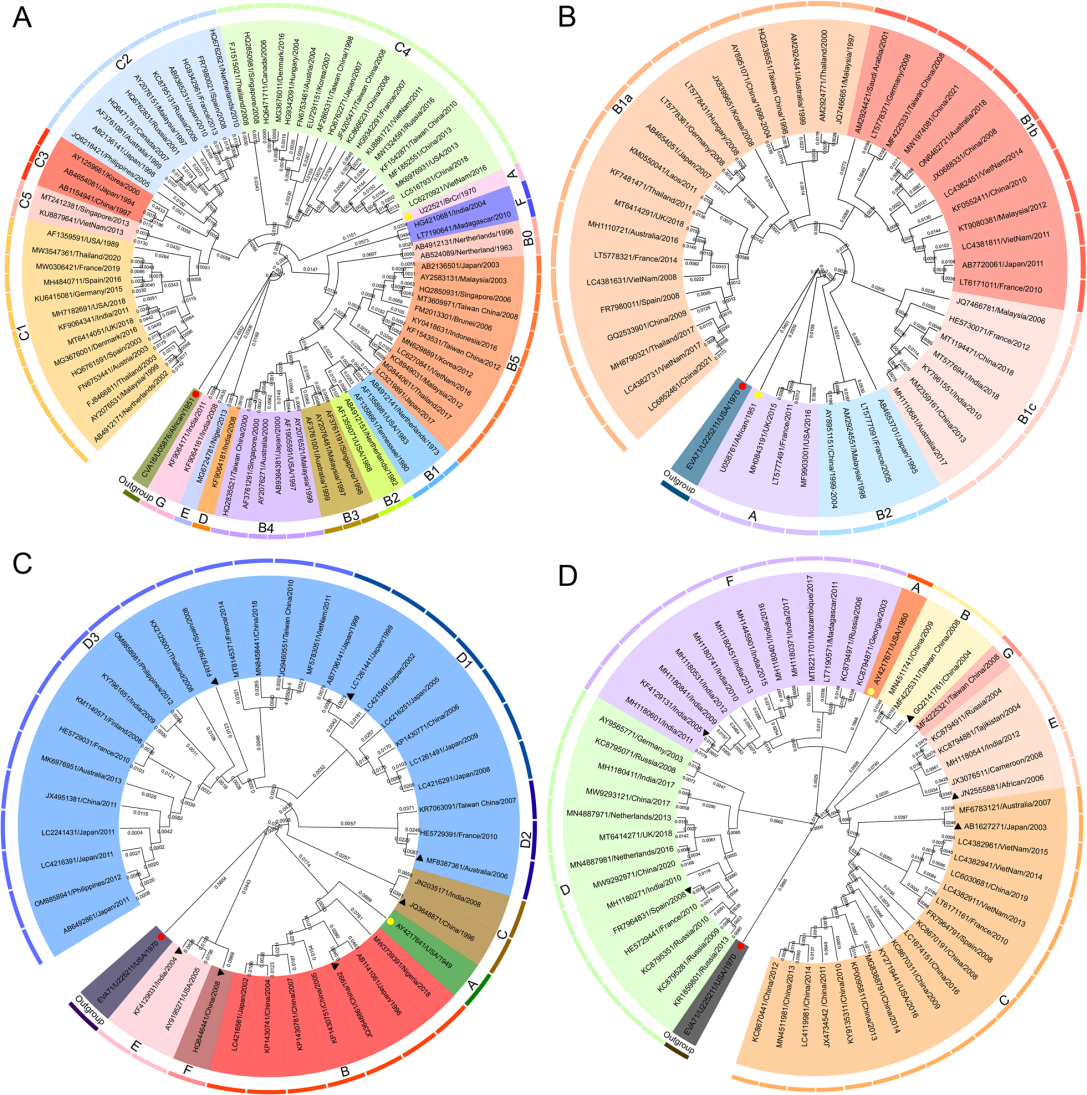
Phylogenetic analyses of the Enterovirus. Phylogenetic analyses of the EV-A71 (**A**), CVA16 (**B**), CVA6 (**C**) and CVA10 (**D**) circulating globally based on full length sequence of the VP1 gene worldwide available from GenBank were conducted in MEGA 7 using the neighbor-joining method. The bootstrap test was performed with 1000 replications. The evolutionary distances were written on the branch. We selected the representative VP1 sequences (EV-A71, n = 84; CVA16, n = 47; CVA6, n = 39; and CVA10, n = 56) from GenBank according to the country of origin, year of isolation and other information. All the strains are labelled using the following format: ‘accession number’/ ‘country of origin’/ ‘year of isolation’. All selected representative strains are marked with distinct colors according to different genotypes/sub-genotypes. The prototypes strains marked with yellow circles and red circles indicate the outgroups. The genotyping reference strains of different genotypes/sub-genotypes of CVA6 and CVA10 are marked as black triangles

EV-A71, CVA16, CVA6 and CVA10 are the 4 major EVs that cause HFMD worldwide. There are no standardized criteria for the classification of subtype, and the different studies on the prevalent types of HFMD use distinct system of sub‐type classification [111]. Bayesian phylogenetic methods with an integrated molecular clock were introduced a decade ago and provided unprecedented opportunities for phylogenetic analysis. In the Review, a difference of at least 15% in the entire VP1 nucleotide sequences is used to distinguish genotypes (Fig. 4) [112]. The sequences were used to identify the serotypes/sub-genotypes using the online Enterovirus Genotyping Tool (http://www.rivm.nl/mpf/enterovirus/typingtool) or a BLAST search. In the case of CVA6 and CVA10, we consult other studies to ensure that the selected strains are representative [110, 113]. The genetic evolution of EV-A71 virus can be divided into seven genotypes (A-G), with genotypes B and C further divided into sub-genotypes B0-B5 and C1-C5, respectively [112]. Genotype A includes the prototype strain (BrCr) isolated in 1969 [5]. C4 and C1 sub-genotypes have developed into the most predominant strain and sub-genotypes C4 circulate mainly in eastern and southeast Asia, whereas C1 are prevalent in Europe [64, 114]. D-G genotypes are relatively rare strains and have been identified in India [115], Africa [116] and Madagascar [117]. There are also several strains that can’t be typed in the online enterovirus Genotyping Tool (defined by some scholars C0: AF135934.1, H: ON646273.1). CVA16 is divided into 2 genogroups A and B with genogroup B being further divided into B1 and B2. Sub-genotype B1 can be further divided into clusters B1a, B1b, and B1c. B1a and B1b can be found in China, Malaysia, Thailand, Australia, Vietnam, and France, Japan et al., which indicate that they co-evolve and co-circulate all over the world [118, 119]. Recently, new genogroups (C and D named by some scholars) have been reported in Peru, France, and China [120–122]. Our results revealed that CVA6 strains could be divided into 6 genotypes designated as A to F, and D genotypes could be further subdivided into D1-3 sub-genotypes. In recent years, the D genotype, particularly D3 sub-genotype, has become the dominant sub-genotype circulating in Southeast Asia and Europe [20, 123]. CVA10 is assigned into 7 genogroups, including genogroup A to genogroup G. Genogroup A is the prototype Kowalik strain isolated in 1950 in the United States [124]. Genogroup B, mainly consisted of CVA10 in China, is assigned to genogroup G. Genogroup C and D include isolates from Russia, Viet Nam, France, America, as well as the latest isolates from Mainland China, which is the predominant circulating strain worldwide [110]. Genogroup E and F mainly circulate in India [125] and Russia [126]. A study on the prevalence of HFMD-associated EVs in China found that more than 98% of EV-A71 sequences belonged to the C4 sub-genotype, with the EV-A71-C4.1 strain having the largest proportion, the longest epidemic period, and the widest geographical distribution. The most predominant strain of CVA16 was CVA16-B1.1, which was widely found in East, Southern, and Northern China. Approximately 95.6% of CVA6 strains belonged to the D genotype and were mainly prevalent in the Eastern, Northern, and Southern regions of China. Most of the CVA10 strains in China belonged to the C sub-genotype and were mainly found in Eastern China [20]. Furthermore, recombination events between other EVs and increased detection rates of these EVs in HFMD samples have been a significant factor in recent HFMD outbreaks. A study of the genome sequence of a novel CVB2 (YN31V3) associated with HFMD found that YN31V3 was likely a recombinant, closely related to CVB2 strains and other EV-B strains [127]. The phylogenetic analysis of CVB3 sequences form the China national HFMD surveillance and global surveillance showed multiple recombination events were present among CVB3 strains circulating globally [128]. Taken together, for evolutionary pressure and frequent recombination, the pathogens of HFMD have evolved into a variety of EVs genotypes with specific temporal and spatial distributions, and further genomic analysis and continuous molecular epidemiological surveillance are helpful for disease control and prevention.

## Pathogenesis

The Viral receptors play a crucial role in the initial stage of infection. The first requirement for virus entry is to bind to the appropriate receptors on the host cells surface, triggering the next step of endocytosis. The availability of receptors often restricts viral infection and influence tissue and species specificity [129, 130]. Currently, most of receptors for EVs belong to the immunoglobulin superfamily (IgSF), which are type I transmembrane glycoproteins [131]. As summarized in Table 2, human scavenger receptor class B member 2 (hSCARB2) [132], P-selectin glycoprotein ligand-1 (PSGL-1) [133], Annexin II [134], Heparan sulfate [135] are identified to be the main receptors of EV-A71, and KREMEN1 was confirmed as a host entry receptor for CVA2, CVA3, CVA4, CVA5, CVA6, CVA7, CVA10, CVA14, CVA16 [136, 137]. EVs interact with host-encoded counterpart receptors and then undergo uncoating, pore formation, and release their genome into the cytosol [138]. EV-A71 binds to hSCARB2, and triggers a clathrin- and dynamin-dependent endocytosis to facilitate viral entry [139]. hSCARB2 and KREMEN1 bind to the canyons at the adaptor-sensor region of EV-A71 and CVA10, respectively, which can also facilitate viral entry [137, 140]. hSCARB2 also induces EV-A71 uncoating under acidic conditions [140–142]. Additionally, the human tryptophan-tRNA synthetase (hWARS) induced by interferon (IFN)-γ has also been recognized as a crucial factor in the entry of EVs [143]. The diversity of receptors and various modes of binding promote EVs infection.

**Table 2:** Major receptors for EVs

Enterovirus	Receptors				
	hSCARB2	PSGL-1	Annexin II	Heparan sulfate	KREMEN1
EV-A71	+	+	+	+	−
CV-A2	−	−	−	−	+
CV-A3	−	−	−	−	+
CV-A4	−	−	−	−	+
CV-A5	−	−	−	−	+
CV-A6	−	−	−	−	+
CV-A7	+	−	−	−	+
CV-A8	−	−	−	−	+
CV-A10	−	−	−	−	+
CV-A12	−	−	−	−	+
CV-A14	+	−	−	−	+
CV-A16	+	−	−	+	+

Human intestinal cells permit infection by EVs such as CVB3 and EV-A71, and can facilitate their replication and release [144]. EV-A71 infects the intestinal epithelium through the apical surface, with a preference for infecting goblet cells. hSCARB2, expressed as an integral membrane protein in goblet cells and localized in intracellular vesicles, provides the necessary condition for viral infection [145]. Although intestinal epithelium induces type IFNs secretion to limit viral replication, viral infection reduces the expression of goblet cells-derived mucins, and alters goblet cell function [146]. Therefore, the targeting of goblet cells by EV-A71 for intestinal infection is likely driven by the enrichment of hSCARB2 in secretory vesicles within these cells, which exposes the receptor through apical mucus release [146]. It is possible that EVs attach to the apical surface using SA glycoproteins and SA-containing glycolipids with SA-linked glycans or dependent decay accelerating factors [147, 148]. Moreover, the tonsillar crypt squamous epithelium, which supports active viral replication, is also an important site for EV-A71 invasion and replication, and is an important source of viral shedding in blood [149]. EVs that invade host cells rapidly complete the viral life cycle (Fig. 2). Subsequently, the virus is released from host cells through a traditional cytolytic manner, and packaged within exosomes, which promote virus spread without causing cell lysis [150, 151]. EVs replicate profusely in cells at the initial site of infection, and then spread to adjacent lymphoid tissues, and next spread to the circulation and target tissues, eventually developing varying degrees of viremia [152]. The proportion of cases with HFMD suffering from viremia is correlated with the duration of complications. In patients with mild HFMD, viremia that occurs improves as symptoms diminish [153]. If viral replication and transmission are controlled at this stage, most infected children will be asymptomatic. However, higher viral loads lead to the development of HFMD as long as the viral infection in the host continues to develop [154]. Together, the virus replicates in the gut early in the infection, and then spreads to the spinal cord, brain, and muscles later in the infection [155]. A part of patients with HFMD develop into more serious complications, including encephalitis, aseptic meningitis, acute flaccid paralysis, and cardiopulmonary failure [156, 157]. The central nervous system (CNS) damage is very common in severe HFMD cases complicated with encephalitis, aseptic meningitis [156]. Clinical reports and animal necropsy studies related to HFMD have revealed the presence of EV antigens in neurons at various locations within the CNS. This suggests that the virus may invade the CNS by compromising the blood–brain barrier (BBB), traveling backwards along nerves, or hijacking immune cells as a means of transportation [152, 158, 159]. Among them, retrograde axonal transport is currently considered as the main pathway for the EVs to invade CNS [160, 161]. Ohka et al. have confirmed through experiments in microfluidic devices that hSCARB2 is necessary for the retrograde axonal transport of EV-A71 [162]. Autopsy pathology revealed significant perivascular intussusception, infiltration of inflammatory cells into the parenchymal, and microglial nodules in the affected CNS. This may have been caused by EVs entering the CNS and infecting neurons, glial cells, the brain stem, the dentate nucleus, and the hypothalamus, ultimately leading to nerve damage [159].

### Innate immune evasion by EVs

**Fig. 5:**
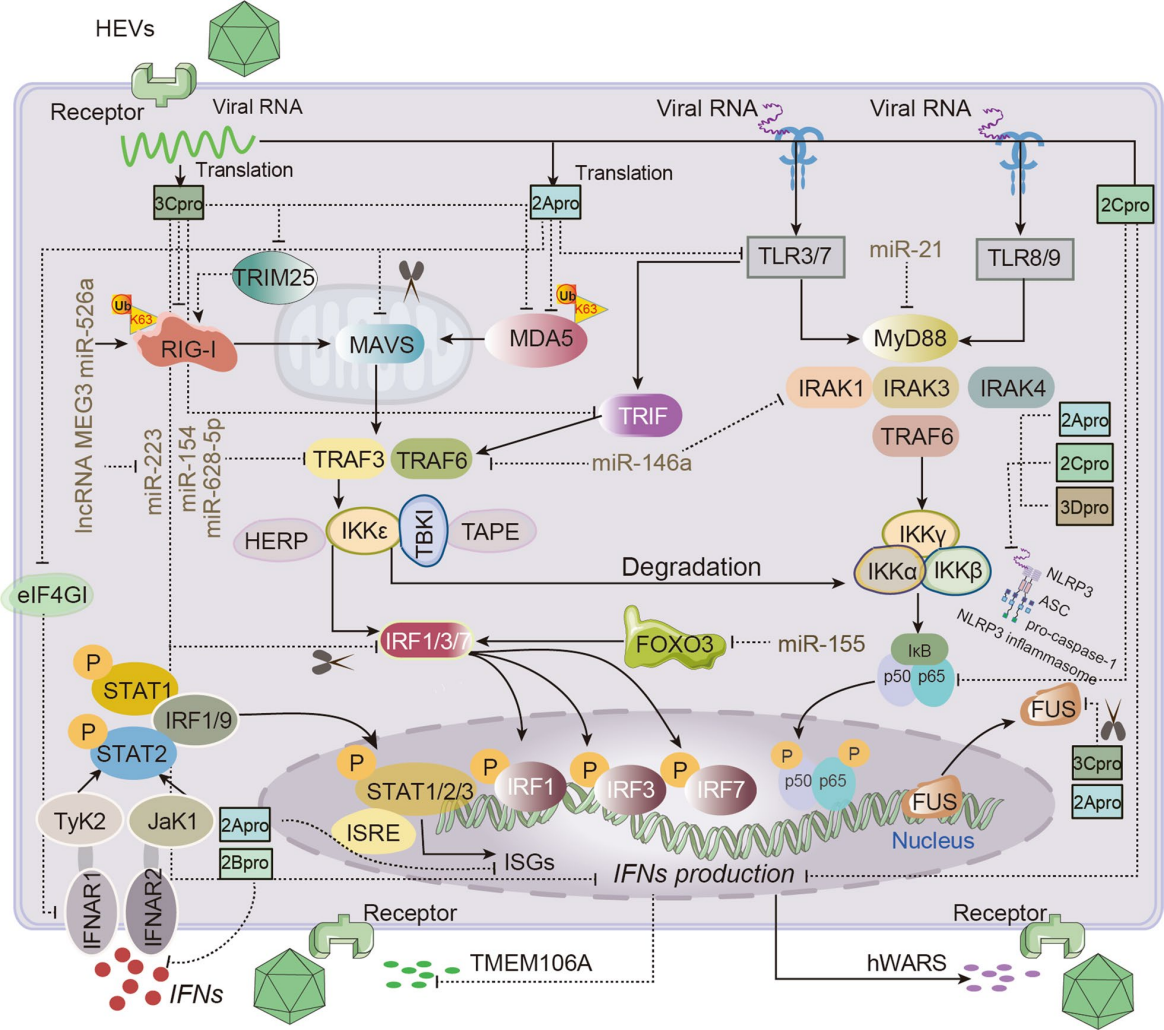
**Innate immune evasion by Enterovirus.** ssRNA, dsRNA and various proteins of EVs during replication and translation can activate and escape innate immunity through different pathways. (1) Viral RNA is recognized by TLR3, TLR7, TLR8 and TLR9, and then activates TRAF, TRIF, MyD88 and their downstream linker molecules, causing phosphorylation of IRF3, IRF7 and NF-κB to translocate to the nucleus, and finally promote the secretion of interferons (IFNs). (2) 2A protease(pro) and 3Cpro are mainly recognized by RIG-I and MDA5, and bind to MAVS in mitochondria to activate TRAF3 and TRAF6. However, prior to signaling to IRF1, IRF3, and IRF7, host ncRNAs regulated by the virus target and inhibit the activation of TRAF, ultimately reducing IFNs production. (3) Binding of IFNs to the receptor IFNAR activates downstream JAK1 and Tyk2, which promotes the phosphorylation and translocation of STAT1 and STAT2 to the nucleus, initiating transcription of IFN-stimulated response elements (ISREs). However, this pathway is directly or indirectly inhibited by 3Cpro, 2Apro, and 2Bpro, resulting in decreased secretion of IFNs. (4) Assembly of the NLRP3 inflammasome requires the sensor NLRP3, the adaptor protein ASC, and pro-caspase-1. However, host-invading viruses can activate and inhibit the formation of the NLRP3 inflammatory complex. Solid line with arrows at the end indicates activation; dashed line with a small line at the end indicates inhibition; scissor indicates cutting

The initial defense against virus is to activate the secretion of IFNs and other antiviral molecules at the site of infection, and to exert their antiviral effects through both autocrine and paracrine mechanisms. The host cell recognizes pathogen-associated molecular patterns (PAMPs) through three pathogen recognition receptors (PRRs): toll-like receptors (TLRs), retinoic acid-inducible gene (RIG-I)-like receptors (RLRs) and nucleotide-binding oligomerization domain (NOD)-like receptors (NLRs) (Fig. 5) [163]. It was discovered that the TLR7, TLR3 and TLR9 can recognize the single-stranded RNA (ssRNA) and double-stranded RNA (dsRNA) of EVs, which then triggers the recruitment of the toll interlukin-1 receptor (TIR). These leads to the activation of the Toll/IL-1R domain-containing adapter-inducing IFN-β (TRIF), which in turn brings in MyD88 into endosomes to further activate innate immune response [164–167]. Other findings suggest that ssRNA and dsRNA are also recognized by the RLR, specifically through the interaction of RIG-I and melanoma differentiation-associated gene 5 (MDA5) with mitochondrial antiviral-signaling protein (MAVS) to activate TANK-binding kinase 1 (TBK1) /IKK-ɛ and IKK-α/β/γ. The phosphorylation of TBK1 activates interferon regulatory factor 3 (IRF3) and stimulates the transcription of IFNs genes [168]. NLRP3 (NOD-, LRR- and pyrin domain-containing 3), as a common inflammasome, has also been demonstrated to play a role in the innate immune response to EVs infection [169]. Additionally, some antiviral molecules could enhance the secretion of IFNs, such as ATP1B3, zinc-finger antiviral protein (ZAP) [170, 171]. There are other unknown pathways for EVs to activate the innate immune response. For example, RNA-binding proteins (RBPs) FUS/TLS inhibited viral replication by interacting with EVs RNA, mediating the formation of SGs and promoting the production of antiviral proinflammatory cytokines and IFN-I [172]. IFNs also directly exert antiviral effects and indirectly induce the transmembrane protein TMEM106A to interfere with the binding of viruses to receptors to reduce cell damage [173, 174].

EVs have developed various tactics to suppress the antiviral response that is mediated by IFNs [163], and the primary impediment to the antiviral pathway is located prior to the production of IFNs. 2Apro and 3Cpro directly inhibit the production of IFNs and the expression of IFNs receptors [175]. EVs mainly act on a variety of protein molecules in the PRR signaling pathway to complete immune evasion. EVs mainly inhibit TLR-dependent signaling mainly by controlling the level of host non-coding RNA (ncRNA) to indirectly influence the TLRs’ ability to sense the host cell, as well as cleaving the downstream molecules MyD88 and TRIF to prevent the IFNs production[176, 177]. 3Cpro could also directly target those key proteins of TLR signaling pathway to inhibit IFNs production [178]. EVs primarily counteract the RLR signaling pathway mainly by directly or indirectly cleaving RIG-I and MDA5, and targeting downstream linker molecules, such as MAVs [179–181]. Current evidence supports the conclusion that EV-A71’s 3D RNA polymerase directly interacts with NLRP3 to form a “3D-NLRP3-ASC” ring structure, which promotes the assembly of the NLRP3 inflammasome complex and the secretion of IL-1β [169]. Meanwhile, EV-A71 2Apro and 3Cpro also cleave NLRP3 to counteract inflammasome activation and inhibit IL-1β secretion [182]. In addition, the IFNs and JAK/STAT signaling pathway are also a key step to further expand antiviral immunity [183]. However, EV-A71 2Bpro and 2Apro selectively target the interferon receptor (IFNAR) directly or indirectly, suppressing the nuclear translocation of STAT1/ STAT2 and the level of ISGE, ultimately limiting the performance of IFNs [184–188]. In addition to participating in common innate immune signaling pathways, EVs also directly inhibit antiviral protein molecules like ZAP and acyl-CoA oxidase 1 (ACOX1). They also indirectly target ubiquitinated key proteins, such as Ubc6e, to induce apoptosis and autophagy, which ultimately exacerbates viral infection [171, 189–191].

### Adaptive immunity to EVs and HFMD

Adaptive immunity also evolved to provide a broader and more sophisticated recognition mechanism to eliminate viruses [192]. Clinical evidence suggests that EVs could elicit neutralizing antibody (NAb) against homotypic viruses [193]. The NAb titers in the serum samples of infected children, collected one day after the symptoms appeared, were more than three times higher than those in healthy children, with the peak occurring at second day [194]. The results of a study investigating the kinetics of EV-A71 NAb response in patients with HFMD showed that the NAb titers rapidly reached a peak within 2 weeks of onset and remained at high levels for 2 years [195]. The study have shown that the serum immunoglobulin IgM (g/L) level of neonatal patients complicated with encephalitis is significantly higher than that of neonatal with lower neurological score [196]. A significant increase in serum and spinal cord IgM and IgG was also observed in EV-A71-infected mice [167]. However, another study found that there is no significant difference between the NAb titers in serum of patients with different severity of HFMD [197]. However, further research is needed to determine the relationship between the antibody response and the severity of HFMD in patients. Chang et al. believe that it is the cellular immunity response rather than the humoral immunity that has a greater impact on determining the outcome of the EV-A71 infection [197]. The cellular immunity carried out by T cells is essential for maintaining body defense. The autopsy biopsy showed abnormal changes in CD4^+^T cells and CD8^+^T cells [198]. Lymphocyte subsets displayed in peripheral blood samples from children infected with EV-A71 showed a decrease in the total number of Th (helper T cell), Tc (killer T cell) and Treg cells (regulatory T cell), and an increase in the percentage of B cells, Th2 and Th17 cells [199]. Furthermore, Th1/Tc1 and Th17/Treg were significantly increased in children infected with mild and severe HFMD [200, 201]. The fast and efficient immune response is a solid line of defense against viral infection. However, an excessive and dysregulated immune response can trigger a series of chain reactions, mainly manifested as systemic immune dysregulation with a neurogenic component.

### Cytokine profiles in HFMD

The levels of cytokines were found to be significantly different among healthy individuals, those with mild HFMD and those with severe HFMD, suggesting a critical role in the progression of the disease and providing potential targets for diagnosis and treatment [202]. Several studies have summarized the cytokines and chemokines associated with severe HFMD, including TNF-α, IFN-γ, IL-1β, IL-18, IL-33, IL -37, IL-4, IL-13, IL-6, IL-12, IL-23, IL-27, IL-35, IL-10, IL-22, IL-17F, IL-8, IP-10, MCP-1, G-CSF, and HMGB1 [202–204]. The innate cells that are involved in cytokine production include neutrophils, macrophages, and natural killer (NK) cells. Meanwhile, and adaptive cells that are involved in ‘cytokine storm’ are mainly various of subsets T cells [205, 206]. The systemic inflammation is usually associated with the breakdown of BBB that accompanies CNS injury, which leads to the entry of brain-derived proinflammatory cytokines into the circulation, further activating the inflammatory cascade including complement [207]. For example, among the lymphocyte chemokines detected, high levels of interferon-gamma-inducible protein-10 (IP-10) were found in the plasma and cerebral spinal fluid of patients with severe HFMD [208]. Additional experiments revealed that a deficiency in IP-10 significantly reduced the levels of Mig (monokine induced by IFN-γ) in serum, and levels of IFN-γ and the number of CD8^+^ T cells in the mouse brain, This, in turn, resulted in an increase mortality rate of EV-A71-infected mice [209]. In addition, the chemokine (C-X-C motif) ligand (CXCL)10 was dramatically upregulated in EV-positive meningoencephalitis group [210]. Our previous study suggested that CXCL10 was highly expressed in the vital organs (brain, lung, heart, and skeletal muscle) of CV-A2-infected mice. Further interference with the CXCL10/CXCR3 axis was found to reduce the levels of leukocytes, neutrophils, and macrophages in the organs of mice that were critically ill [211]. Critical HFMD patients showed a decreased in peripheral blood lymphocytes, a depletion of CD4^+^and CD8^+^T lymphocytes, and a decline cellular immunity [212]. Ultimately, the immune system collapses and multiple organs of the host are damaged, leading to irreversible multi-organ failure and death.

### Mechanisms of neurological damage and cardiopulmonary failure

Fatal complications of infections affecting the nervous system are directly or indirectly linked to damage of nerve cells. EV-A71 3Cpro directly cleaves the host DNA repair enzyme poly (ADP-ribose) polymerase and induces apoptosis [213]. EV-A71 3Dpro indirectly induces apoptosis and inflammation by downregulates ACOX1 expression and promotes reactive oxygen species (ROS) generation [191, 214]. The CNS damage is not only related to viral replication, but frequently associated with immune activation [215]. Recent discoveries indicate that nerve cells that express TLR7, TLR3, TLR8, and TLR9 can rapidly induce the secretion of IFNs in response to infection with EVs, which provides antiviral protection [164–167]. For instance, EV-A71 triggers a response in glia cells that involves the production of Interleukin-12p40 through TLR9 signaling, leading to the generation of neurotoxic Inducible Nitric Oxide Synthase (iNOS)/Nitric Oxide (NO), resulting in encephalitis [216]. In addition, the Janus kinase (JAK)-signal transducer of activators of transcription (STAT) pathway also regulates the expression of IFNs [184, 187]. Conversely, the virus antagonizes the antiviral response of nerve cells by cleaving RIG-I, which further inhibits the JAK/STAT signaling pathway [217, 218].

PE is one of the most serious complications of HFMD aside for encephalitis, and is the primary reason of rapid death of patients with severe HFMD [212, 219]. The development of PE is closely linked to inflammation in the CNS and ‘cytokine storm’ that is triggered by abnormally high depletion of IL-10, IL-13, IFN-γ and a depletion of lymphocyte in plasma [207, 212]. The currently recognized pathogenesis of fulminant PE is neurogenic [219–222]. Autopsy results showed extensive inflammatory in the CNS with severe PE [223]. A clinical study in Taiwan showed a significant correlation between CNS involvement and PE in children infected with EV-A71 [219]. Similarly, acute PE caused by Japanese encephalitis is associated with disruption of the anti-hypertensive mechanisms in the medulla of the CNS [224]. In the development of HFMD, damage to CNS leads to immune disorders, which are primarily manifested by the excessive release of catecholamines and cytokines [8, 207, 212]. Wu et al. proposed that the further increase in pulmonary vascular permeability caused by inflammatory response is the underlying cause of PE [225]. The CVA6-infected mice and EV-A71-infected hSCARB2 KI mice exhibited significant PE and hemorrhage, with the infiltration of neutrophil and monocyte in brain and spinal cord [226, 227]. In CVA2-infected mouse model, endothelial dysfunction, local inflammation, and enhanced vascular permeability were confirmed to be involved in accelerating acute lung injury [228]. Cardiac damage caused by EVs mainly progresses to acute heart failure (AHF) and myocarditis. During the HFMD outbreak in Taiwan in 1997, some severe patients presented with AHF [229]. The main cause of AHF in patients is acute left ventricular dysfunction and regional wall motion abnormalities [230]. The underlying cause of cardiac damage may be hypercatecholamineremia caused by brainstem encephalitis, which further leads to the cardiotoxicity of AHF [231, 232]. Myocardial cell necrosis is rarely observed in cardiac autopsy of EV-A71-infected patients [221]. In recent years, the emerging CVB3 among children infected with EVs has garnered increased attention [233]. As the most common pathogen causing viral myocarditis, CVB3 seems to promote cardiac function damage mainly by inducing myocardial apoptosis and necrosis [234]. Despite Lucie et al. not providing a comprehensive explanation of a fatal case of CVA2-related myocarditis in France, our colleagues noticed significant inflammatory and swelling in the heart of a mouse model infected with CVA2 [235, 236].

## HFMD treatment

Unfortunately, there are currently no established antiviral treatments for HFMD and no specific clinical management and treatment methods have been established. For common cases, general treatment is usually used, isolating patients to avoid cross-infection, and taking good oral and skin care to avoid contamination. According to the development of HFMD, the treatment corresponding to the intervention of critical patients usually includes antiviral therapy, immunoglobulin therapy, respiratory and circulatory system support, etc.

### Antiviral therapy

IFN-α, and ribavirin treatment have shown positive effect in antiviral management of HFMD to some extent [237, 238]. Various drugs, like antiviral peptides, small molecules, have been identified promising candidates, but their full pre-clinical validation have yet to be reported [239, 240].

### Intravenous immunoglobulin (IVIG)

In previous outbreak of HFMD, IVIG was used on a presumptive basis for the treatment of severe cases [223, 241–243]. Recently, some anecdotal evidence suggests that the use of IVIG in the early stage of HFMD can significantly improve the progression of the disease and reduce mortality [244, 245]. Compared with conventional therapy alone, conventional therapy combined with IVIG had shorter fever clearance time, shorter rash regression time, and shorter clinical cure time [246].

### Respiratory support

Mechanical ventilation is the most effective treatment to improve oxygen supply to the body [247]. The application of indications and the withdrawal indications are described in Chinese guidelines for the diagnosis and treatment of HFMD (2018) [21]. If they occur seizures (frequent myoclonic jerks), routine anti-convulsant may be considered, such as sedation (e.g., midazolam) and/or anticonvulsants (e.g., phenytoin).

### Treatment of catecholamine storm

Early application of esmolol can effectively stabilize the vital signs of severe HFMD by reducing serum catecholamine concentration, alleviating myocardial damage, improving cardiac function, and reducing inflammatory response. The phentolamine can reduce mortality and relieves the symptoms of EV-A71-induced PE, which is a potential therapeutic agent for neurogenic PE [248].

### Cardiovascular support

Multiple inotropes to support cardiac function (milrinone, dobutamine, dopamine, epinephrine) have been applied in the clinical treatment [247]. Among them, Milrinone exhibits immunoregulatory and anti-inflammatory effects in the management of systemic inflammatory response in severe cases [249]. If the above drugs prove ineffective, vasopressin or levosimendan can be considered [21].

### Intracranial pressure control

Mannitol is commonly used to reduce increased intracranial pressure, the combination with hypertonic saline or diuretics may be considered for patients with severe intracranial hypertension [21, 250].

### Traditional Chinese medicine

The combined Chinese medicine and chemistry medicine therapy achieve a better therapeutic efficacy in the treatment of severe HFMD than the chemistry medicine therapy alone [251]. The addition of Andrographolide Sulfonate and S. baicalensis to conventional therapy also reduces the occurrence of major complications, relieves fever, and attenuates oral lesions and rashes [252, 253].

### Others

A retrospective observational study showed that continuous veno-venous hemodiafiltration could improve cardiovascular function [254]. Extracorporeal life support, including extracorporeal membrane oxygenation (ECMO), is last rescue treatment for patients who have failed to routine symptomatic and supportive treatment [8, 21].

Taken together, the main approach to treating severe cases of HFMD is mainly through supportive and symptom-relieving measures. There is a need to carry out more clinical studies to gather more evidence to improve the clinical management of severe cases.

## Long-term sequelae of HFMD

Severe HFMD occurs mainly affects preschool children under the age of 5, a crucial stage in their growth and development. Although treatment advancements have led to a decrease in acute mortality, there are still concerns about the potential possible short-term or long-term impacts (Fig. 1).

### Neurological dysfunction

A substantial burden of neurological sequelae following HFMD has been given more attention, especially in severe cases [6, 255, 256]. Among patients who experienced cardiopulmonary failure after CNS involvement, the proportion with subsequent sequelae (facial nerve palsy, limb weakness and atrophy, dysphagia, central hypoventilation, seizure, and psychomotor retardation) was significantly higher compared to those who only CNS involvement. The clinical severity of CNS involvement was significantly related to the children’s neurodevelopment (a delay in the gross motor and personal-social categories, delayed neurodevelopment) [257–260]. Serious virus-associated CNS infection during childhood appear to be associated with the later mental disorders, like attention-deficit hyperactivity disorder (ADHD) diagnosis alongside social/communication/emotion problems and autistic features [261–263]. Some severe EV-A71 infected patients may experience impaired speech and language skills due to subcortical white matter involvement in the acute stage [258–260]. Long-term functional neurological morbidity is associated with the involvement of medulla oblongata, gray matter in the brainstem or spinal cord, which may be closely monitored for early intervention and meticulous management [258, 264, 265].

### Visual impairment

HFMD-related eye involvement presents variable signs, including pseudomembranous conjunctivitis [266], outer retinitis [267] and maculopathy [268], which is only observed in young adult patients in both sexes and always unilaterally. Despite self-limited nature and complete visual recovery in most cases later than resolution of HFMD symptoms (several weeks to months), some cases may have residual visual loss.

### Nail abnormalities

Delayed skin and nail change, such as desquamation of palms and soles [269, 270], Beau’s lines, or onychomadesis [271], have also been observed in some severe EV-A71 infected patients. Nail change, mainly presenting as onychomadesis involving toenails or fingernails, is usually observed among 1–2 months after the onset of HFMD and lasted for 1–8 weeks, most for approximately 4 weeks and the changes are more likely to occur synchronously [272]. It can occur in both children and adults [273, 274]. The pathogens associated with nail abnormalities in HFMD patients are various, but mainly caused by CVA6 [269]. Nail change is usually self-limited with spontaneous healed requiring no treatment for all patients [275, 276].

In addition to focusing on the common health effects of HFMD, other health problems should not be ignored. Allergic diseases: a population-based cohort study has revealed that children suffered from HFMD had decreased risks of asthma [277]. In contrast, another retrospective cohort study found that the risk of asthma was higher in children with herpangina and HFMD [278]. Diabetes: One adult patient with severe atypical HFMD associated with CVA6 viremia showed impaired glucose tolerance after 2-year follow-up [279]. Heart diseases: A population-based cohort study has showed meningitis caused by herpangina/HFMD is the main disease associated with a higher risk of Kawasaki disease [280]. Idiopathic ventricular tachycardia, degenerative aortic valve disease, degenerative mitral valve disease, may be considered as sequelae of CVA6 infection in adults [279]. Nephropathy: A large national cohort study showed that children infected with EVs, particularly coxsackieviruses, had a significantly increased risk of developing nephrotic syndrome [281]. Leukemia: The risk of leukemia was significantly lower in the EVs-infected cohort, and herpangina/HFMD was the main disease reduced the risk of leukemia [282]. Long-term follow-up programs are crucial for early recognition of possible sequelae and early intervention in children who have suffered from HFMD, especially at a young age. Further studies are needed to better understand the pathogenesis of HFMD and its impact on sequelae.

## Vaccine development

Vaccination is considered the most effective and cost-effective approach to control the incidence of HFMD. Currently, there are monovalent and polyvalent vaccines available against the HFMD pathogen. The monovalent vaccines consist mainly of inactivated whole virus vaccines, synthetic peptide and protein vaccines [283], recombinant subunit vaccines [284], and recombinant virus-vector vaccine [285]. Currently, the most readily available inactivated whole virus vaccines for EV71 are produced by Sinovac, Vigo, and the Chinese Academy of Medical Sciences (CAMS). Results from a randomised, double-blind phase 3 trial in China showed that the inactivated EV71 vaccine has a 97.4% efficacy rate [286]. The monovalent inactivated virus vaccine candidates for CVA16, CVA10, CVA6, and CVA5 have only been studied in animal models and lack clinical evidence of protection [287–290]. However, the limited scope of protection offered by monovalent vaccines, which are specific to one genotype, means that they do not provide protection against other EVs-associated cases of HFMD. Therefore, the most effective approach for reducing the incidence of HFMD is to use polyvalent vaccines that have been developed through the combination of effective monovalent vaccines or by constructing chimeric vaccines with different virus serotypes, which can provide better cross-reactivity and protection. Polyvalent vaccines, which aim to improve cross-reactivity, consist mainly of inactivated polyvalent vaccines, polyvalent virus-like particle vaccines, innovative chimeric vaccines, and recombinant virus-vector vaccines. Currently, the inactivated polyvalent vaccines, including bivalent, trivalent, and quadrivalent vaccines, have mainly been tested for their protective effects in animal studies. Vaccines formulated by combining inactivated EV-A71 and CVA16 viruses induced specific immunity against EV-A71 and CVA16 infections in animal models [291, 292]. The CVA6 and CVA10 inactivated whole virus bivalent vaccines have been shown to elicit high levels of neutralizing antibodies in mice [293]. The induction of a strong neutralizing antibody response and cell-mediated immune response was also shown to occur with the administration of inactivated whole virus trivalent vaccines [294, 295]. The antigen-specific and persistent serum antibody responses by quadrivalent vaccines were comparable to those by the respective monovalent vaccines [296]. In addition, polyvalent virus-like particles, novel chimeric vaccines, and recombinant virus-vector vaccines have all shown to induce broad protective effects and enhance systemic immune responses [297]. Antigenic peptide-based vaccine development and DNA/RNA vaccine technology be also applied for future exploration of polyvalent vaccines [298, 299]. However, it is important to carefully consider the inclusion of appropriate strains and to thoroughly evaluate the immunogenicity and immune interactions when developing multivalent vaccines.

## Surveillance

The World Health Organization (WHO) primarily manages the existing global surveillance network for poliovirus, but has not yet established a specialized network to monitor HFMD or EVs. The National Enterovirus Surveillance System (NESS), established in the United States as a passive and laboratory-based system, has been used to track EVs reports since the 1960s, and provides the most comprehensive data for monitoring HFMD [300]. The Asia–Pacific Network for Enterovirus Surveillance (APNES) was established in 2017 through collaboration between academic institutions and hospitals in the Cambodia, Malaysia, Vietnam, and Taiwan region [301]. However, the efficiency of the system is limited due to its limited coverage and the absence of a unified governing body. In 2008, HFMD was incorporated into China’s notifiable infectious disease reporting system. In order to better prevent and control HFMD, China has gradually established and improved a nationwide monitoring network system for HFMD laboratories, with prefecture-level laboratories, provincial-level laboratories, and national-level laboratories as the main body. Most European countries have established national surveillance systems for laboratory-based detection of EVs. Currently, the Prospective, Multicenter and Cross-sectional Hospital Pilot Non-Polio Enterovirus Network program, which was jointly established by several European countries, is set to become operational in 2022 [302]. Laboratory-based disease surveillance networks can result in inefficient use of limited typing resources. Therefore, more optimized monitoring programs have been developed and applied to estimate HFMD incidence and optimize serotype estimation [303]. However, based on epidemiological data from dynamic surveillance of EVs that may cause HFMD, there has been a rise in the incidence of HFMD associated with some non-EV-A71/CVA EVs infections [304]. In recent years, the increasing occurrence of multiple EV infections and novel patterns of recombinant EV infections in patients with HFMD highlights the need for more vigilant pathogen surveillance of HFMD, especially in regards to emerging and co-infected pathogens [305].

## Conclusions

In this Review, we systematically summarize the current knowledge on virology, epidemiology, pathogenesis, long-term sequelae of HFMD. Finally, as we assemble and interpret this evolving knowledge base, we need to understand which approaches to prevention and treatment, in this context, are most feasible and cost-effective, requiring a concerted effort between basic medical researchers and pediatricians. Overall, our study provides all relevant knowledge and the latest progress of HFMD, which will better inform health care and policy.

## Data Availability

All relevant data are within the manuscript and its additional files.

## Abbreviations

HFMD: Hand-foot-and-mouth disease
CFDA: China Food and Drug Administration
CV: Coxsackievirus
Echo: Echoviruses
EVs: Enteroviruses
PV: Poliovirus
ORF: Open reading frame
PE: Pulmonary edema
COVID-19: Coronavirus disease
hSCARB2: Human scavenger receptor class B member 2
PSGL-1: P-selectin glycoprotein ligand-1
BBB: Blood–brain barrier
PAMPs: Pathogen-associated molecular patterns
PRRs: Pathogen recognition receptors
TLRs: Toll-like receptors
RIG-I: Retinoic acid-inducible gene
RLRs: RIG-I-like receptors
NOD: Nucleotide-binding oligomerization domain
NLRs: NOD-like receptors
ssRNA: Single-stranded RNA
dsRNA: Double-stranded RNA
TIR: The toll interlukin-1 receptor
TRIF: The Toll/IL-1R domain-containing adapter-inducing IFN-β
MDA5: Melanoma differentiation-associated gene 5
MAVS: Mitochondrial antiviral-signaling protein
TBK1: TANK-binding kinase 1
IRF3: Interferon regulatory factor 3
ZAP: Zinc-finger antiviral protein
RBPs: RNA-binding proteins
ncRNA: Non-coding RNA
IFNAR: IFNs receptor
ACOX1: Acyl-CoA oxidase 1
NAb: Neutralizing antibody
NK: Natural killer
IP-10: Interferon-gamma-inducible protein-10
CXCL: The chemokine ligand
ROS: Reactive oxygen species
JAK: Janus kinase
STAT: Signal transducer of activators of transcription
AHF: Acute heart failure
IVIG: Intravenous immunoglobulin
ECMO: Extracorporeal membrane oxygenation
ADHD: Attention-deficit hyperactivity disorder
CAMS: The Chinese Academy of Medical Sciences
NESS: National Enterovirus Surveillance System
WHO: The World Health Organization
APNES: The Asia–Pacific Network for Enterovirus Surveillance
